# Cost-effectiveness of longer-term versus shorter-term provision of antibiotics in patients with persistent symptoms attributed to Lyme disease

**DOI:** 10.1371/journal.pone.0195260

**Published:** 2018-04-02

**Authors:** Anneleen Berende, Lisette Nieuwenhuis, Hadewych J. M. ter Hofstede, Fidel J. Vos, Michiel L. Vogelaar, Mirjam Tromp, Henriët van Middendorp, A. Rogier T. Donders, Andrea W. M. Evers, Bart Jan Kullberg, Eddy M. M. Adang

**Affiliations:** 1 Department of Internal Medicine, and Radboud Center for Infectious Diseases, Radboud University Medical Center, Nijmegen, Netherlands; 2 Department for Health Evidence, Radboud University Medical Center, Nijmegen, Netherlands; 3 Department of Epidemiology, CAPHRI—School for Public Health and Primary Care, Maastricht University, Maastricht, Netherlands; 4 Sint Maartenskliniek, Nijmegen, Netherlands; 5 Department of Medical Psychology, Radboud University Medical Center, Nijmegen, Netherlands; 6 Institute of Psychology; Health, Medical, and Neuropsychology Unit, Leiden University, Leiden, Netherlands; TNO, NETHERLANDS

## Abstract

**Background:**

The treatment of persistent symptoms attributed to Lyme disease remains controversial. Recently, the PLEASE study did not demonstrate any additional clinical benefit of longer-term versus shorter-term antibiotic treatment. However, the economic impact of the antibiotic strategies has not been investigated.

**Methods:**

This prospective economic evaluation, adhering a societal perspective, was performed alongside the PLEASE study, a multicenter, placebo-controlled, double-blind 1:1:1 randomized clinical trial in which all patients received open-label intravenous ceftriaxone for two weeks before the 12-week randomized blinded oral antibiotic regimen (doxycycline, clarithromycin plus hydroxychloroquine, or placebo). Between 2010 and 2013, patients (n = 271) with borreliosis-attributed persistent symptoms were enrolled and followed for one year. Main outcomes were costs, quality-adjusted life years, and incremental net monetary benefit of longer-term versus shorter-term antibiotic therapy.

**Results:**

Mean quality-adjusted life years (95% CI) were not significantly different (p = 0.96): 0.82 (0.77–0.88) for ceftriaxone/doxycycline (n = 82), 0.81 (0.76–0.88) for ceftriaxone/clarithromycin-hydroxychloroquine (n = 93), and 0.81 (0.76–0.86) for ceftriaxone/placebo (n = 96). Total societal costs per patient (95% CI) were not significantly different either (p = 0.35): €11,995 (€8,823-€15,670) for ceftriaxone/doxycycline, €12,202 (€9,572-€15,253) for ceftriaxone/clarithromycin-hydroxychloroquine, and €15,249 (€11,294-€19,781) for ceftriaxone/placebo. Incremental net monetary benefit (95% CI) for ceftriaxone/doxycycline compared to ceftriaxone/placebo varied from €3,317 (-€2,199-€8,998) to €4,285 (-€6,085-€14,524) over the willingness-to-pay range, and that of ceftriaxone/clarithromycin-hydroxychloroquine compared to ceftriaxone/placebo from €3,098 (-€888-€7,172) to €3,710 (-€4,254-€11,651). For every willingness-to-pay threshold, the incremental net monetary benefits did not significantly differ from zero.

**Conclusion:**

The longer-term treatments were similar with regard to costs, effectiveness and cost-effectiveness compared to shorter-term treatment in patients with borreliosis-attributed persistent symptoms after one year of follow-up. Given the results of this study, and taking into account the external costs associated with antibiotic resistance, the shorter-term treatment is the antibiotic regimen of first choice.

## Introduction

Lyme borreliosis, a tick-borne disease caused by the spirochete *Borrelia burgdorferi* sensu lato complex, is the most common tick-borne disease in the northern hemisphere and its incidence has been increasing considerably in several countries worldwide [1, 2]. Patients in the early stages of Lyme disease can often be treated successfully with antibiotics [3, 4]. However, regardless of initial appropriate antibiotic treatment, persistent symptoms may develop that consist of pain, neurologic or cognitive impairments, musculoskeletal symptoms and/or fatigue [5].

The disease burden of Lyme disease is large, and the disability-adjusted life years (DALYs) per 100,000 population were estimated at 10.55 in 2010, resulting in 1749 DALYs for the Dutch population [6]. Mainly persistent symptoms are a considerable source of healthcare utilization and costs [6–8]. Since the incidence of Lyme disease is rising in several countries, there are concerns that the significant economic and disease burden of persistent symptoms attributed to Lyme disease will increase further.

The treatment of persistent symptoms attributed to Lyme disease remains controversial, as previous trials found inconclusive results [9–11] and as clinical guidelines recommend different treatment durations [12–14]. Recently, the Persistent Lyme Empiric Antibiotic Study Europe (PLEASE), which evaluated the effectiveness of longer-term versus shorter-term antibiotic treatment among patients with borreliosis-attributed persistent symptoms, did not demonstrate any additional clinical benefit of longer-term antibiotic treatment compared to shorter-term treatment [15].

Regardless of clinical effect, it is important to assess the economic impact of the comparative antibiotic strategies. This is essential for policy makers, in order to prioritize and making complex decisions about healthcare interventions. Therefore, we performed the first cost-utility analysis of longer-term versus shorter-term provision of antibiotics in patients with persistent symptoms attributed to Lyme disease.

## Materials and methods

### Study design and patients

This economic evaluation was performed alongside the PLEASE study, a multicenter, placebo-controlled, double-blind randomized clinical trial, which was conducted in the Netherlands at the Radboud university medical center and the Sint Maartenskliniek (ClinicalTrials.gov number NCT01207739). Its design and main results have been described in detail elsewhere [15, 16]. Briefly, patients were included if they experienced persistent symptoms attributed to Lyme disease, such as pain, musculoskeletal symptoms, neuralgia, sensory disturbances, arthritis, arthralgia, or neuropsychological/cognitive complaints, with or without persistent fatigue. These symptoms had to be preceded by an erythema migrans (EM) or otherwise confirmed symptomatic Lyme disease, or patients were required to have *B*. *burgdorferi* IgG or IgM antibodies. After inclusion, patients were randomly allocated in a 1:1:1 ratio to three treatment arms. All patients received 2000 mg open-label intravenous ceftriaxone every day for two weeks before starting the blinded oral antibiotic regimen of 12 weeks. The randomized oral treatment consisted of 100 mg of doxycycline twice daily combined with a placebo twice daily, 500 mg clarithromycin twice daily combined with 200 mg of hydroxychloroquine twice daily, or two placebos twice daily. Ethical clearance of the PLEASE study protocol was obtained from the Medical Ethics Review Committee CMO Region Arnhem-Nijmegen and all patients provided written informed consent before inclusion.

### Outcome measures

The main outcome measures of the economic evaluation were costs and EQ-5D-based quality-adjusted life years (QALYs) [17]. These outcome measures were combined into the incremental Net Monetary Benefit (NMB), adhering a societal perspective, over a one-year follow-up period. The societal perspective includes the impact of an intervention on the welfare of the whole of society, by including not only direct health effects (both costs and QALYs) but also indirect health effects (such as productivity losses) [18, 19]. Outcomes were assessed at baseline and at 14, 26, 40 and 52 weeks follow-up by self-completed questionnaires.

#### Effectiveness

The quality of the health status of the patients was measured with a validated health-related quality of life (HRQoL) instrument, the EuroQol-5D (EQ-5D) [20]. This HRQoL instrument was offered in a validated Dutch translation and was completed by the patients at all evaluation moments [17]. The EQ-5D is a generic HRQoL instrument comprising five domains: mobility, self-care, usual activities, pain/discomfort and anxiety/depression. The EQ-5D index is obtained by applying predetermined weights to the five domains. This index gives a societal-based global quantification of the patient’s health status on a scale ranging from zero (death) to one (perfect health). Based on the EQ-5D index scores, QALYs were determined over the total follow-up period by using the trapezium rule to estimate the area under the curve.

#### Costs

The following cost categories were considered: intervention costs, healthcare utilization, pain medication utilization, travel expenses and productivity losses. Intervention costs were standardized and, for each patient, consisted of one hospital admission day for the first ceftriaxone administration, two weeks of outpatient treatment with ceftriaxone, 12 hours of home care, and 12 weeks of treatment with the randomized oral regimen. The cost analysis comprised two main parts. First, on a patient level, volumes of care were measured prospectively over the time path of the clinical trial using an adapted version of the first part of the 'Trimbos and iMTA questionnaire on Costs associated with Psychiatric illness' (TIC-P)[21] complemented with patient out-of-pocket expenses for pain-related over-the-counter drugs. Where relevant, (missing) entries were verified or completed by data from medical records. Second, standard cost prices were determined using the Dutch guideline for cost analysis in healthcare research [22] and www.medicijnkosten.nl, a website managed by the Dutch National Health Care Institute, for drug prices per daily dose. If no standardized cost prices were available, real tariffs or costs were used. Productivity losses for patients were estimated using a patient-based questionnaire (Short Form–Health and Labor Questionnaire (SF-HLQ)) [23]. The friction cost-method was applied in accordance with the Dutch guidelines [22]. In addition, travel expenses were computed for every healthcare visit based on the patient’s postal code and the location of healthcare provision. Data on resource use were multiplied by standardized unit prices to calculate costs in the treatment groups. Costs were calculated in Euros using 2015 as base-year value. Prices were indexed using the Consumer Price Index (CPI) from Statistics Netherlands (CBS).

### Data analysis

Patients who had been randomized into the study and who had received at least one dose of ceftriaxone were included in the modified intention-to-treat analysis. If patients only completed baseline assessment, they were considered uninformative and therefore excluded from the economic analysis. In the base-case analysis, missing data regarding QALYs and costs were imputed with the nearest available observation as this was considered the most realistic scenario. Missing data regarding time between follow-up visits were imputed a single time using a uniform random number generator, which was limited by the minimum and maximum in the available data.

Analyses of covariance, with paid work and baseline values as covariates, were used to compare mean costs and mean QALYs gained between the three treatment groups. The cost-effectiveness analysis comprised computing NMBs for each patient by multiplying the QALYs that were gained during the one year follow-up with a range of ‘willingness-to-pay (WTP) for a QALY’ thresholds and then subtracting the total costs from this amount. Subsequently, the incremental NMBs were calculated by subtracting the mean NMB of the placebo group from the mean NMBs of the longer-term treatment arms. The NMB framework, combined with multivariable linear regression, was used to correct for relevant baseline differences (paid work), baseline EQ-5D index score and baseline total costs. Because the cost-effectiveness threshold in the Netherlands ranges from €10,000 to €80,000 per QALY depending on the disease burden [24, 25], six thresholds for the maximum WTP for a QALY were applied ranging from zero to 100,000 Euros. For all regression models, 1,000 bootstrap replications were used to account for skewness of the distribution of the estimator (NMB) in order to obtain robust 95% confidence intervals of the estimates. IBM SPSS Statistics 22.0 was used as statistical software package.

#### Imputation scenario analyses

To analyze the effect of the abovementioned missing data imputation strategy on our results, two other imputation scenarios for missing QALYs and costs were applied in addition to the more realistic base-case nearest available observation imputation: as a best-case scenario EQ-5D index scores were imputed with the 75^th^ percentile of the available data and the total costs with the 25^th^ percentile, and as a worst-case scenario EQ-5D index scores were imputed with the 25^th^ percentile and total costs with the 75^th^ percentile of the available data.

## Results

In total, 281 patients were enrolled into the PLEASE study between October 2010 and June 2013, and followed for one year. Of these, 271 patients were included in the cost-utility analysis: one patient did not start the ceftriaxone treatment; nine other patients only underwent baseline EQ-5D assessment. Baseline characteristics were not significantly different between the study groups, except for paid work, gaining income from the Work and Income according to Labor Capacity Act (WIA), and travel expenses in the three months before the start of the study (Table 1).

**Table 1:** Baseline characteristics.

	Ceftriaxone + doxycycline (N = 82)	Ceftriaxone + clarithromycin-hydroxychloroquine (N = 93)	Ceftriaxone + placebo (N = 96)	P value^a^
Age—mean (SD)	48.6 (12.8)	48.5 (13.1)	50.2 (9.7)	0.56
Female sex–no. (%)	38 (46)	41 (44)	46 (48)	0.87
Employment status^b^ –no. (%)				
Paid work	49 (60)	65 (70)	76 (79)	0.02
Unpaid work	7 (9)	6 (7)	7 (7)	0.87
Unemployed	4 (5)	2 (2)	2 (2)	0.47
Student	3 (4)	5 (5)	2 (2)	0.49
Housewife/man	14 (17)	15 (16)	14 (15)	0.90
General old-age insurance	11 (13)	8 (9)	8 (8)	0.46
Sickness Benefits Act	17 (21)	20 (22)	27 (28)	0.43
Labor disability (WIA)	17 (21)	15 (16)	6 (6)	0.02
EQ-5D index score–mean (SD)	0.58 (0.26)	0.59 (0.25)	0.64 (0.23)	0.22
Direct costs within healthcare^c^ –mean (SD)	23 (39)	36 (89)	15 (26)	0.05
Pain medication (€)	7 (19)	5 (10)	6 (13)	0.68
Healthcare consumption (€)	497 (785)	1292 (5611)	582 (2074)	0.25
Direct costs outside healthcare^c^ ^–^mean (SD) Travel expenses (€)	23 (39)	36 (89)	15 (26)	0.05
Indirect costs^c^ –mean (SD) Productivity losses (€)	2544 (5535)	1972 (4824)	2710 (4660)	0.57
Total costs^c^ (€)–mean (SD)	3072 (5770)	3036 (7328)	3314 (5078)	0.96

^a^ Continuous variables were compared between the groups by using an ANOVA, categorical variables by using chi-squared tests. Not normally distributed data were analyzed with a bootstrapped ANOVA.

^b^ Categories are not mutually exclusive.

^c^ Baseline costs are the costs in Euros made in the three months before the start of the study. WIA, Work and Income according to Labor Capacity Act.

**Fig 1:**
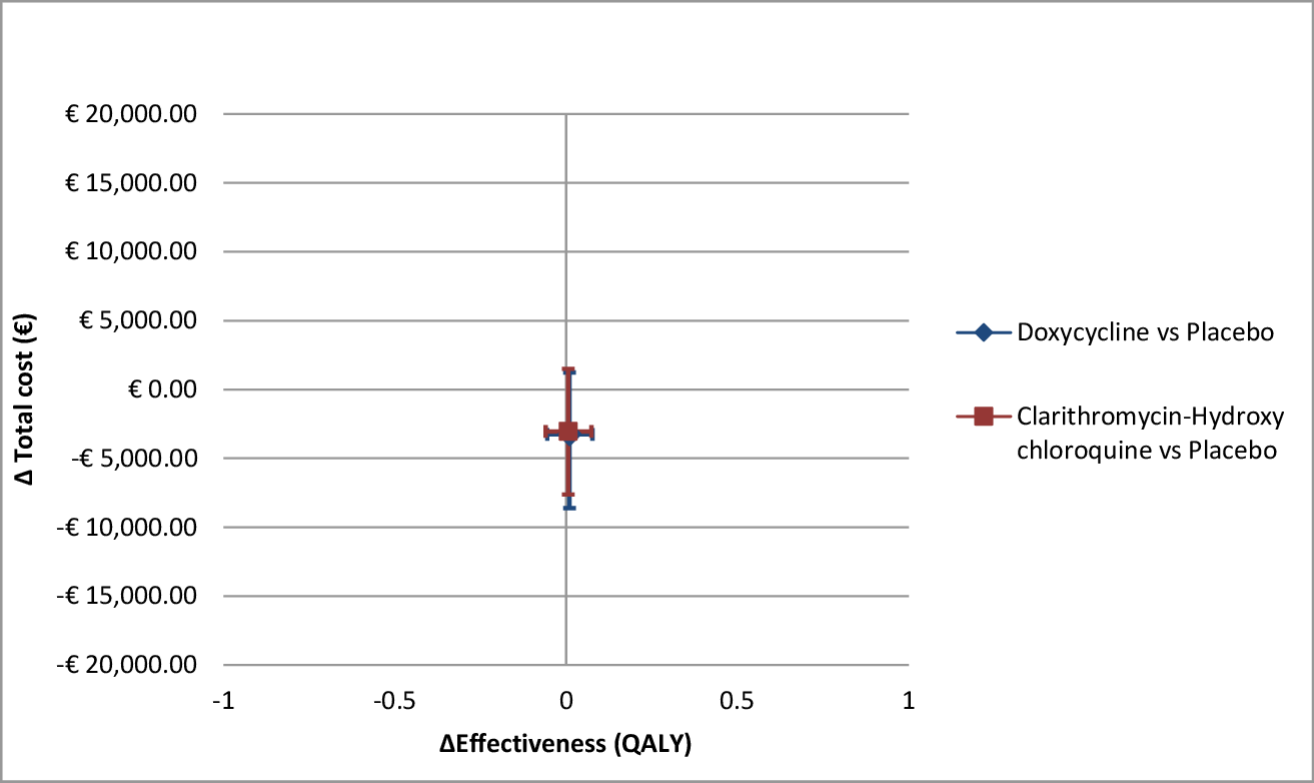
Incremental total costs plotted against incremental QALYs with 95% confidence intervals.

The average amount of QALYs yielded during the one-year follow-up period was 0.82 (95% CI, 0.77–0.88) for the ceftriaxone plus doxycycline group, 0.81 (95% CI, 0.76–0.88) for the ceftriaxone plus clarithromycin-hydroxychloroquine group, and 0.81 (95% CI, 0.76–0.86) for the ceftriaxone plus placebo group. These were not significantly different between the groups (p = 0.96). The mean total societal costs over the one-year study period were also not statistical significantly different between the groups (p = 0.35): €11,995 (95% CI, €8,823-€15,670) for the ceftriaxone plus doxycycline group, €12,202 (95% CI, €9,572-€15,253) for the ceftriaxone plus clarithromycin-hydroxychloroquine group and €15,249 (95% CI, €11,294-€19,781) for the ceftriaxone plus placebo group. The incremental total costs were plotted against the incremental QALYs for each longer-term treatment group compared to the placebo group in Fig 1.

Antibiotic therapy, productivity losses and healthcare consumption were the main cost drives in all study groups (Table 2). No significant differences in mean costs between the study arms were found in any of the cost categories, although the point estimates for healthcare consumption varied considerably (Table 2).

**Table 2:** Mean QALYs and costs (in euro’s) per patient over the 1-year follow-up period.

	Ceftriaxone +doxycycline (N = 82)		Ceftriaxone + clarithromycin-hydroxychloroquine (N = 93)		Ceftriaxone +placebo (N = 96)		P value^a^
	Mean	(95% CI)	Mean	(95% CI)	Mean	(95% CI)	
QALYs	0.82	(0.77–0.88)	0.81	(0.76–0.88)	0.81	(0.76–0.86)	0.96
Direct costs within healthcare							
Antibiotic therapy^b^	2254	(-)	2282	(-)	2211	(-)	-
Pain medication	36	(23–52)	22	(16–29)	33	(22–45)	0.24
Healthcare consumption	1802	(1211–2517)	2324	(1508–3286)	3296	(1675–5521)	0.35
Direct costs outside healthcare Travel expenses	104	(66–148)	58	(39–75)	83	(51–127)	0.23
Indirect costs Productivity losses	7667	(4466–12039)	7858	(5450–10667)	9392	(6941–12270)	0.70
Total costs	11995	(8823–15670)	12202	(9572–15253)	15249	(11294–19781)	0.28

^a^ Bootstrapped ANCOVA corrected for baseline value and paid work.

^b^ Costs of the antibiotic therapy were standardized.

QALY, quality-adjusted life year.

**Fig 2:**
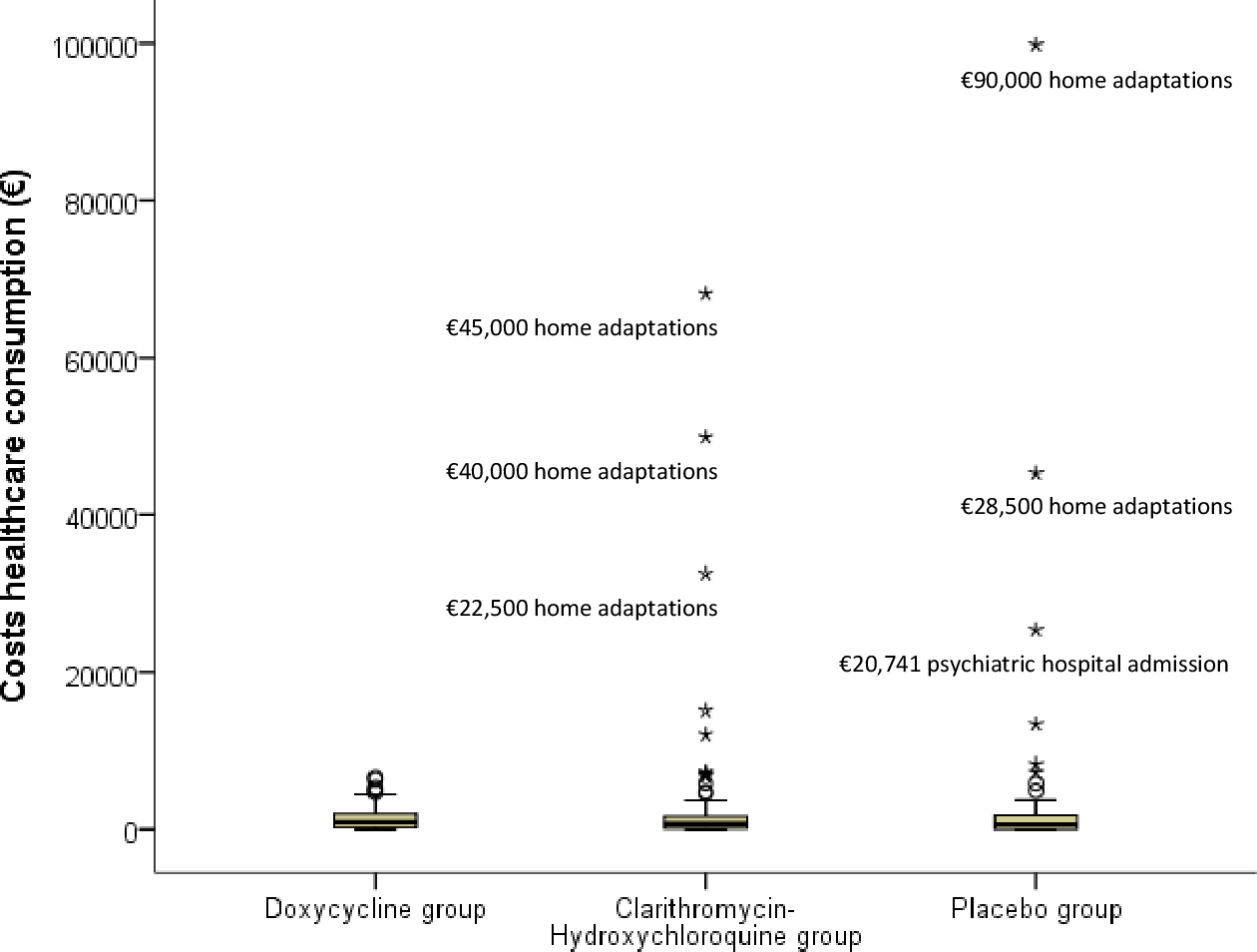
Distribution of the costs of healthcare consumption in the three treatment groups, including explanation of the largest outliers.

Fig 2 shows the distribution of healthcare consumption costs. As shown in Fig 2, a few outliers in the clarithromycin-hydroxychloroquine and placebo groups are responsible for the large distribution width. The outliers were mainly due to high costs of home adaptations (e.g. placement of an elevator). Due to bootstrapping the relevance of these outliers is relatively small and had little influence on the coefficients nor confidence intervals of the NMB regression model.

**Fig 3:**
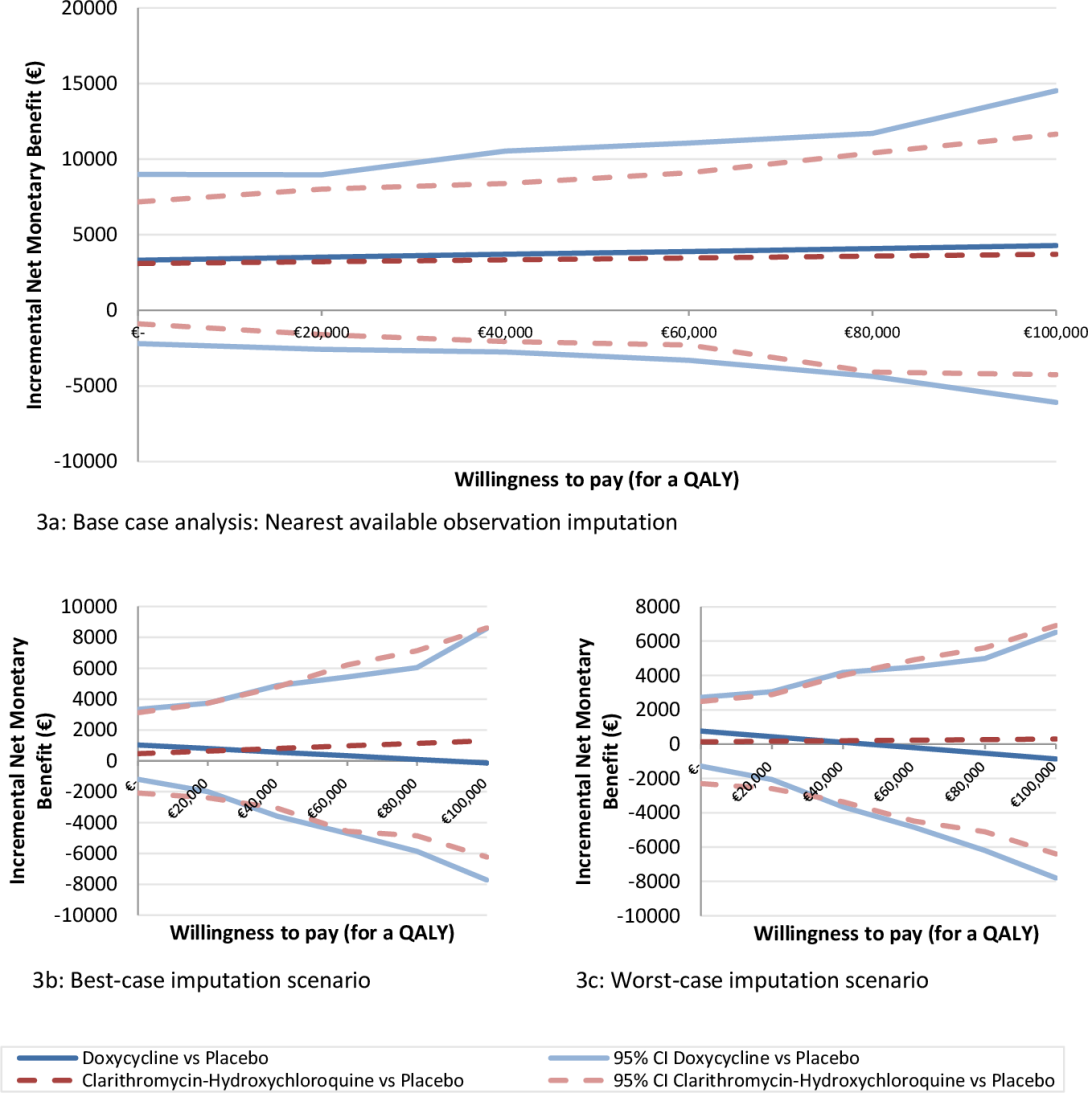
a. Base case analysis: Nearest available observation imputation. b. Best-case imputation scenario. c. Worst-case imputation scenario.

Fig 3A presents the incremental Net Monetary Benefit (iNMB) with 95% CI of both longer-term treatment groups compared to the placebo group over the ‘willingness-to-pay (WTP)’ range from 0 to 100,000 Euros per QALY. The iNMB for the doxycycline group compared to the placebo group varied from €3,317 (95% CI, -€2,199-€8,998) to €4,285 (95% CI, -€6,085-€14,524) over the WTP range, and that of the clarithromycin-hydroxychloroquine group compared to the placebo group from €3,098 (95% CI, -€888-€7,172) to €3,710 (95% CI, -€4,254-€11,651). For all WTP thresholds, the iNMBs did not significantly differ from zero. The imputation scenario analyses showed similar results (Fig 3B and 3C).

## Discussion

This study is the first economic evaluation of antibiotic therapy regimens in patients with borreliosis-attributed persistent symptoms, and was performed alongside the PLEASE study. We found no differences in mean total societal costs and mean QALYs between the three groups after a one-year study period. There was, given the range of evaluation, no willingness-to-pay threshold for which the incremental Net Monetary Benefit of the longer-term treatment groups compared to the placebo group was significantly different from zero. The imputation scenario analysis also showed no significant differences in cost-effectiveness between the groups.

These results are relevant for the care of patients with persistent symptoms attributed to Lyme disease, as they complement the effectiveness knowledge obtained from the PLEASE study and other research. To our knowledge, no other studies have investigated the cost-utility of antibiotic treatment regimens of persistent symptoms attributed to Lyme disease. Because the PLEASE study did not show any additional clinical benefit of longer-term compared to shorter-term treatment on health-related quality of life, we did not expect to find any large differences in costs and cost-effectiveness as the oral antibiotic treatment is low-priced. Nevertheless, because of the limited resources in healthcare, cost-effectiveness of the treatment strategies should be carefully considered as one of the criteria in a rational decision-making process.

Strengths of this economic evaluation include the prospective design, in which data were collected alongside a multicenter, placebo-controlled, double-blind randomized clinical trial. This study was the largest trial that evaluated antibiotic therapies in patients with borreliosis-attributed persistent symptoms. Data on health states and costs were prospectively collected on patient-level, which made it possible to give relatively precise estimates. Moreover, we performed our analyses from a societal perspective, since we included productivity losses and travel expenses as cost categories as well. This is the optimal perspective, as it includes all relevant societal costs and benefits irrespective of who bears or accrues them, and is recommended by the Dutch guideline for economic evaluations in healthcare [22].

Our study also has limitations. First, there were missing data. Missing data are almost unavoidable in economic evaluations performed alongside a clinical trial, especially when patients have to self-report the data and when costs and cost-effectiveness are not the primary outcome measures. To overcome this problem and to assess the effect of our imputation strategy, we imputed our data according to three scenarios. Since all scenarios gave slightly different estimates but similar conclusions, our results are robust against assumptions regarding the missingness of data.

Furthermore, the cause of the persistent symptoms is poorly understood. Consequently, despite our strict inclusion criteria, it is unclear whether all symptoms in our study population are attributable to Lyme disease. Nevertheless, this population represents the patients who are actually encountered in clinical practice and who do suffer from low quality of life, have high healthcare consumption and productivity losses. Therefore, research regarding the effectiveness, costs and cost-effectiveness of treatment regimens in this patient group is essential for clinical practice.

Costs of potential antibiotic resistance among both the patients’ intestinal flora and the environment [26, 27] were not included in our evaluation. If these costs could have been taken into account, the longer-term treatment regimens likely would have been less favorable in terms of costs and cost-effectiveness compared to the shorter-term treatment. Antibiotic resistance is a growing global threat, which has been estimated to cause 23,000 deaths and $55 billion of healthcare costs and productivity losses each year in the United States [28]. In Europe, these numbers were estimated to be 25,000 deaths and €1.5 billion yearly [28]. Since antibiotic resistance is directly related to volumes of antibiotic treatment and since resistance of both first-line and last-resort antibiotics is increasing rapidly, ineffective and even potentially harmful treatment with antibiotics should be prevented.

## Conclusions

From a societal perspective, the longer-term treatments of ceftriaxone combined with doxycycline or with clarithromycin and hydroxychloroquine were as costly, effective and cost-effective as shorter-term treatment with ceftriaxone only in patients with persistent symptoms attributed to Lyme disease after one year of follow-up. Taking into account the growing concern to antibiotic resistance because of unnecessary use, the shorter-term provision of antibiotics should be preferred.

## Supporting information

S1 File Minimal anonymized data set. (SAV)
